# What Are the Factors Associated with the Structural Damage Differences in Open-Angle Glaucoma? RNFL- and GCIPL-Dominant Progression

**DOI:** 10.3390/jcm11226728

**Published:** 2022-11-14

**Authors:** Sung Eun Park, Jihei Sara Lee, Mijung Kim, Chan Yun Kim, Hyoung Won Bae

**Affiliations:** 1 Institute of Vision Research, Department of Ophthalmology, Yonsei University College of Medicine, Seoul 03722, Korea; 2 Department of Medicine, Yonsei University Graduate School, Seoul 03722, Korea

**Keywords:** glaucoma, retinal nerve fiber layer, ganglion cell-inner plexiform layer

## Abstract

We sought to analyze the parameters associated with retinal nerve fiber layer (RNFL)-dominant progression or ganglion cell–inner plexiform layer (GCIPL)-dominant progression in patients with open-angle glaucoma. A prospective observational study was conducted. Overall, 58 eyes from 33 patients with open-angle glaucoma were categorized into the following two groups: patients with RNFL- and GCIPL-dominant progression, and the primary outcome was the difference in associated factors between two groups. Higher pre-treatment and mean IOP, greater lamina cribrosa curvature index (LCCI), and younger age were more significantly associated with the RNFL-dominant progression group than the GCIPL-dominant progression group. When adjusting for mean IOP, age, LCCI, and microvascular dropout (MVD), only pre-treatment IOP was significantly associated with the RNFL-dominant progression group. However, when adjusting for pre-treatment IOP, age, LCCI, and MVD, both higher mean IOP and greater LCCI were significantly associated with RNFL-dominant progression. In conclusion, pre-treatment and mean IOP and LCCI were more strongly associated with the RNFL-dominant progression group than the GCIPL-dominant progression group. In contrast, age, peripapillary choroidal microvascular dropout, and systolic and diastolic blood pressures tended to damage the GCIPL predominantly rather than the RNFL. Therefore, our findings suggest the potential to set different treatment targets and identify various treatment methods for each group.

## 1. Introduction

Glaucoma, which is the most prominent type of acquired optic neuropathy, is characterized by the progressive loss of retinal ganglion cells (RGCs) presenting as structural changes in the optic nerve head (ONH) and retinal nerve fiber layer (RNFL) with corresponding visual field (VF) defects [1]. Glaucoma is a multifactorial optic neuropathy in which both localized ocular factors (intraocular pressure (IOP), RNFL loss, and ganglion cell–inner plexiform layer (GCIPL) loss) and systemic factors (autonomic dysfunction including high or low blood pressure, cold extremities, and sleep apnea) are involved. Although IOP is a substantial risk factor, IOP is not elevated in more than half of the patients with untreated glaucoma [1]. Therefore, defining glaucoma based on characteristics other than IOP is crucial. Several attempts have been made to discriminate normal-tension glaucoma (NTG) from high-tension open-angle glaucoma (OAG). However, the etiology of NTG, which is characterized as IOP within the statistically normal range of <21 mmHg, is yet to be well defined. Therefore, identifying the pathogenic factors and mechanism of glaucomatous optic nerve damage through comparisons among individuals is challenging.

The ability to detect structural loss is fundamental in diagnosing and managing glaucoma [2]. Glaucomatous structural damage, including RNFL and GCIPL thinning and ONH change, can be assessed objectively and quantitatively using optical coherence tomography (OCT) [2]. The evaluation of RNFL and GCIPL loss using OCT instruments is assumed to be helpful in the early diagnosis of the specific type of OAG and proper further management. Kim et al. compared the macular ganglion cell complex thickness of patients with NTG and high-tension OAG. They concluded that the macular ganglion cell complex was more related to patients with NTG than to the high-pressure OAG group. In addition, in high-tension OAG eyes, the lamina cribrosa is located more posteriorly and has a greater curvature relative to NTG and healthy eyes of a comparable age [3].

We have observed that some patients in our clinic undergo RNFL-dominant progression rather than GCIPL-dominant progression, although the opposite is true for other patients. Several studies have attempted to determine the RNFL or GCIPL diagnostic factors; however, no study has compared RNFL- or GCIPL-dominant progression and their related factors. Therefore, we categorized patients with OAG into RNFL- and GCIPL-dominant progression groups and analyzed the parameters affecting these two different groups specifically.

## 2. Materials and Methods

### 2.1. Patient Enrollment

This was a prospective, observational study. In this study, patients with more than 5 years of regular follow-up for OAG before 2021 at Yonsei University Health System and who had either RNFL-dominant or GCIPL-dominant progression were recruited. Patients who visited the Yonsei University Health System between March 2021 and September 2021 were enrolled. In addition, each patient provided written informed consent for participation. The Institutional Review Board of Yonsei University Hospital (IRB number 4-2021-1013) approved this study, which was conducted according to the tenets of the Declaration of Helsinki. As documented by glaucoma experts, all participants in this study had open angles on gonioscopy and glaucomatous changes in the ONH. A systematic evaluation was performed, and data, including medical history, age, sex, systolic and diastolic blood pressure, primary diagnosis, and complete ocular examination, were analyzed. In addition, best-corrected visual acuity, refractive error assessment, slit-lamp examination, Goldmann tonometry, gonioscopy, dilated stereoscopic examination of the optic disc, disc photography, and red-free fundus photography were performed on all participants. Other ocular examinations included scanning of the optic nerve head (ONH) using spectral-domain OCT (Spectralis OCT; Heidelberg Engineering, Heidelberg, Germany), circumpapillary RNFL and GCIPL thickness, and peripapillary choroidal microvascular dropout (pcMvD) using a Zeiss Cirrus 5000 HD-OCT (Cirrus OCT; Carl Zeiss Meditec. Inc., Jena, Germany), standard automated perimetry (Humphrey Field Analyzer II 750 and 24-2 Swedish interactive threshold algorithm; Carl Zeiss Meditec, Dublin, CA, USA), and axial length (AXL) (IOLMaster version 5; Carl Zeiss Meditec. Inc., Jena, Germany). Glaucoma severity was determined by using Hodapp–Parrish–Anderson criteria.

### 2.2. Study Design

Forty-eight patients at Yonsei University Health System with regular follow-ups for more than 5 years before 2021 and having progression on either RNFL or GCIPL were eligible for inclusion. We defined and classified the patients into the following two groups using Cirrus OCT: the RNFL- and GCIPL-dominant progression groups. Patients who showed RNFL-dominant progression in at least two consecutive Cirrus OCT measurements with stable GCIPL progression were classified into the ‘RNFL-dominant progression group’; the opposite cases were categorized into the ‘GCIPL-dominant progression group’. Among the patients that could not attend the clinic, 14 patients canceled appointments due to COVID-19, and 1 patient died of old age. Consequently, 33 patients (58 eyes) who were able visit our clinic from March 2021 to September 2021 were finally enrolled. Subsequently, we compared the following glaucomatous parameters in the two groups with: age, pre-treatment and mean IOP, lamina cribrosa curvature index (LCCI), AXL, pcMVD, choroidal thickness, systolic and diastolic blood pressure, and presence of disc hemorrhage.

### 2.3. Intraocular Pressure Measurements

IOP before initiating ocular hypertensive treatment was obtained through medical record examination and was referred to as pre-treatment IOP. In addition, the mean follow-up IOP measurement was obtained by averaging the IOP measured at 6-month intervals after using IOP-lowering medication.

### 2.4. Enhanced Depth Imaging Optical Coherence Tomography of the Optic Disc

The optic nerve head of each eye was visualized with Spectralis OCT using an enhanced depth imaging technique. In addition, a 10° × 15° rectangle covering the optic disc was used to perform the imaging. This rectangle was scanned with approximately 70 sections, which were 30–34 mm apart (the slicing distance was determined automatically by the machine). The average number of frames per section was 42, which provided the best trade-off between image quality and patient cooperation. All images were post-processed using adaptive compensation to enhance the visibility of the peripheral lamina cribrosa (LC).

### 2.5. Definition and Measurement of the Lamina Cribrosa Curvature Index

The optic nerve was visualized using the enhanced depth-imaging technique of the Spectralis OCT system. In a report by Kim et al., the details and advantages of this technology have been described for evaluating the LC [4]. Briefly, the LCCI was determined by measuring the width of the LC curve reference line (W), which is defined as the width of the line connecting the two points on the anterior LC surface that met the lines drawn from each Bruch’s membrane opening (BMO) termination point perpendicular to the BMO reference line, and LC curve depth, the maximum depth from the reference line to the anterior LC surface. Furthermore, LCCI was calculated as (LC curve depth/W) × 100. Two glaucoma specialists (SEP and HWB), who were blinded to the clinical information, measured and calculated LCCI. Moreover, the mean values determined by the two observers were averaged and analyzed.

### 2.6. Peripapillary Retinal Nerve Fiber Layer and Ganglion Cell–Inner Plexiform Layer Thickness

OCT images were acquired by macular scan (macular cube 200 × 200 protocol) and peripapillary RNFL scan (optic disc cube 200 × 9 × 200 protocol) after pupil dilation using Cirrus OCT (software version 6.0). In addition, the macular GCIPL thickness within a 6 × 6 × 2 mm (14.13 mm^3^) elliptical annulus around the fovea was measured and computed using Cirrus OCT software version 11.5.1. The annulus cube was 1 mm, 4 mm, 1.2 mm, and 4.8 mm for the inner vertical, outer vertical, inner horizontal, and outer horizontal diameters, respectively, excluding the central portions of the fovea where the layers are thin and difficult to defect. The average GCIPL thickness was used for analysis. Furthermore, the peripapillary RNFL thickness within a 3.46 mm diameter circle automatically positioned around the optic disc was measured, and the average RNFL thickness was measured and analyzed. The progression of RNFL or GCIPL defects was estimated using the Cirrus OCT-guided progression analysis (GPA; Carl Zeiss Meditec. Inc., Jena, Germany) program, which aligns and compares changes in RNFL and GCIPL thicknesses between the follow-up and 2 baseline RNFL and GCIPL thickness maps (with at least a 6-month interval in this study). GPA provides color-coded classification in a 6 × 6 mm^2^ (50 × 50 superpixels). When the differences in RNFL and GCIPL thickness between the follow-up and the first- and second-baseline RNFL and GCIPL thickness maps were greater than the test–retest variability, the RNFL and GCIPL thickness change map is shown by yellow. In addition, the existence of the differences in consecutive follow-up visits is shown by red in the RNFL and GCIPL thickness change map. In this study, progressive RNFL and GCIPL thinning was defined when at least 20 contiguous pixels, coded red, in the RNFL or GCIPL thickness change map were detected in at least two consecutive examinations. In addition, only OCT results with a signal strength of 7 or higher were collected.

### 2.7. Definition of Peripapillary Choroid Microvascular Dropout and Choroidal Thickness

The choroidal microvasculature within the peripapillary area is of particular clinical interest since it is downstream of the short posterior ciliary artery, which also perfused the prelaminar tissue and the lamina cribrosa. The presence of the pcMvD was examined using the Zeiss Cirrus 5000 HD-optical coherence tomography angiography (OCTA) (Zeiss Meditec. Inc., Jena, Germany). The choroidal microvasculature was evaluated using 4.5 × 4.5 mm^2^ choroid-disc vessel density maps of the optic nerve head [5]. Capillary dropout in the OCTA image was regarded as pcMvD [5]. In addition, choroidal thickness was measured from Bruch’s membrane to the choroid–sclera interface from the choroidal image using Heidelberg Spectralis OCT.

### 2.8. Statistical Analysis

The glaucomatous parameters (IOP, mean deviation (MD) from Humphrey visual field test, RNFL thickness, GCIPL thickness, pcMvD, AXL, LCCI, disc hemorrhage, and choroidal thickness) were calculated and compared using the Statistical Package for the Social Sciences software, version 26.0 (IBM Corporation, Armonk, NY, USA). Furthermore, differences between the groups were examined using the *t*-test, Fisher’s exact test, linear regression, and logistic regression. Statistical significance was set at *p* < 0.05.

## 3. Results

### 3.1. Baseline Characteristics

The baseline characteristics are presented in Table 1. Overall, 58 eyes from 33 patients with OAG were eligible for inclusion. The RNFL-dominant progression group included 22 eyes from 12 patients, and the GCIPL-dominant progression group included 36 eyes from 21 patients. A medical history of hypertension was observed in 3 (13.64%) and 13 (36.11%) patients in the RNFL- and GCIPL-dominant progression groups, respectively (*p* = 0.063). The mean ± standard deviation age, pre-treatment IOP, mean IOP, AXL, visual field MD, choroidal thickness, LCCI, and mean systolic and diastolic blood pressure are presented in Table 1. After using anti-glaucoma medication, pre-treatment and mean IOP were significantly higher in the RNFL-dominant progression group (*p* = 0.004). In addition, LCCI, which is known to be associated with elevated IOP, was greater in the RNFL-dominant progression group (*p* = 0.031). However, no significant difference was observed in systolic and diastolic blood pressures between the two groups (*p* = 0.151 and 0.806, respectively). Furthermore, pcMvD and disc hemorrhage were not significantly different (*p* = 0.141).

### 3.2. Factors Associated with the Retinal Nerve Fiber Layer-Dominant Progression Group

**Figure 1:**
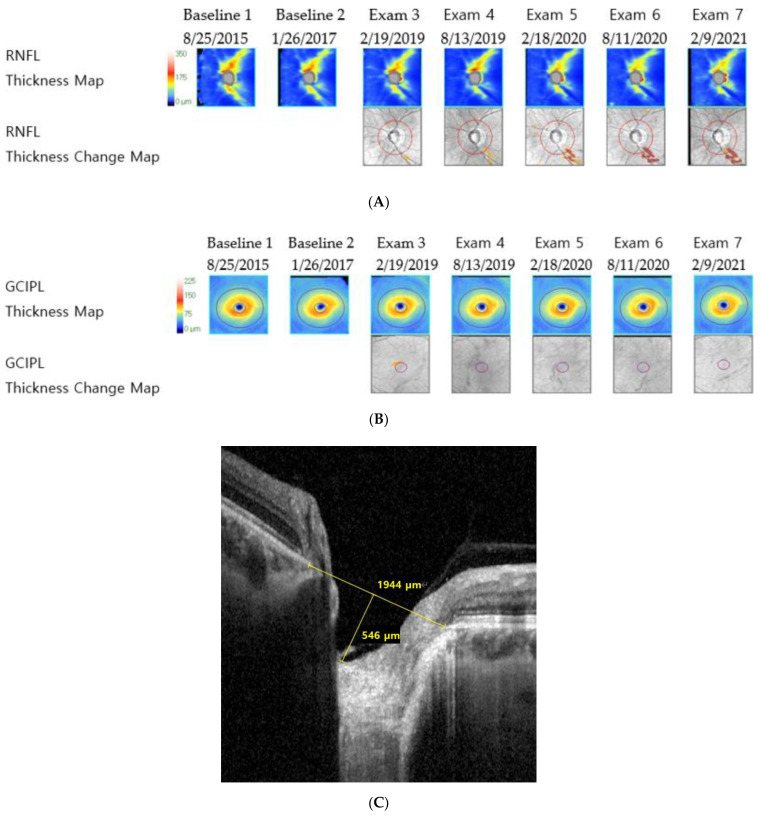
Representative images of a patient with primary open-angle glaucoma from the retinal nerve fiber layer-dominant progression group (mean intraocular pressure with Goldmann tonometry: 17.33 mmHg, axial length: 22.66 mm, visual field mean deviation: −4.3 dB). (**A**) Retinal nerve fiber layer deviation and thickness-guided progression analysis from Cirrus optical coherence tomography shows progressive retinal nerve fiber layer thinning, (**B**) ganglion cell–inner plexiform layer deviation and thickness-guided progression analysis from the Cirrus optical coherence tomography shows no progression, and (**C**) infrared image of lamina cribrosa from Spectralis optical coherence tomography shows large lamina cribrosa curvature index.

The results of the univariate and multivariate conditional logistic regression analyses assessing factors associated with the RNFL-dominant progression group are presented in Table 2. The univariate analysis revealed that higher pre-treatment IOP (odds ratio [OR], 1.292; 95% confidence interval [CI] = 1.057, 1.580; *p* = 0.012) and mean IOP (OR, 1.522; CI = 1.066, 2.053; *p* = 0.019), greater LCCI (OR, 1.070; CI = 1.004, 1.140; *p* = 0.037), and younger age (OR, 0.962; CI = 0.925, 1; *p* = 0.049) were significantly associated with RNFL dominant progression. Notably, only pre-treatment IOP (OR, 1.325; CI = 1.058, 1.661; *p* = 0.014) was significantly associated with the RNFL-dominant progression group when adjusting for mean IOP, age, LCCI, and pcMvD (Table 2, multivariate analysis 1). However, higher mean IOP (OR, 1.655; CI = 1.139, 2.405; *p* = 0.008) and greater LCCI (OR, 1.094; CI = 1.007, 1.188; *p* = 0.035) were significantly associated with the RNFL-dominant progression group when adjusting for pre-treatment IOP, age, LCCI, and pcMvD (Table 2, multivariate analysis 2). Figure 1 illustrates a representative case from the RNFL-dominant progression group, with progressive RNFL defects, stable GCIPL thickness, and relatively large LCCI.

### 3.3. Factors Associated with the *Ganglion Cell–Inner Plexiform Layer* Dominant Progression Group

**Figure 2:**
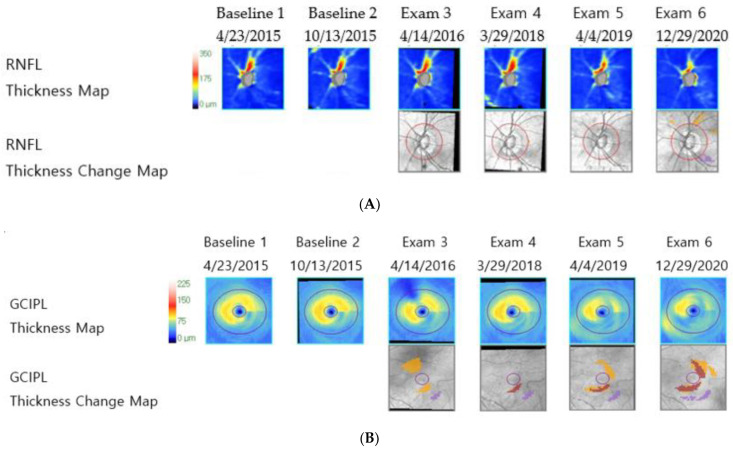

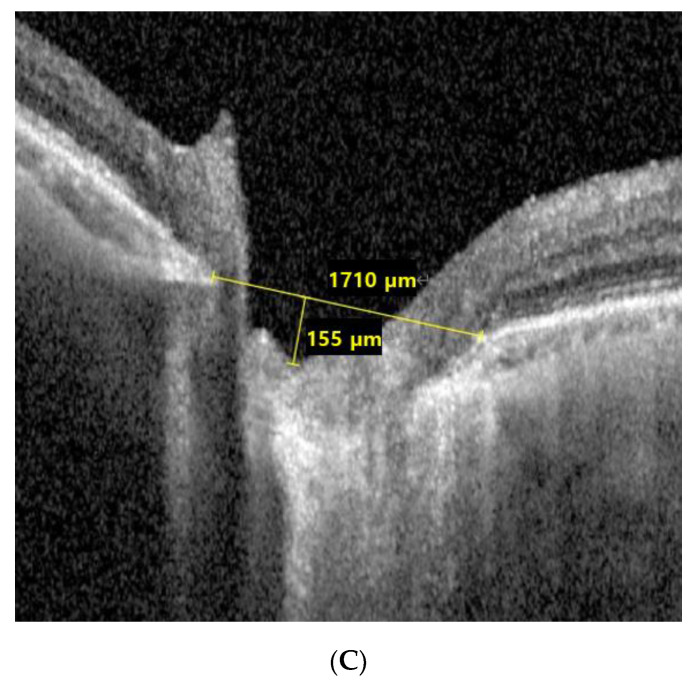
Representative images of a patient with normal-tension glaucoma from the ganglion cell–inner plexiform layer dominant progression group (mean intraocular pressure with Goldmann tonometry: 13.18 mmHg, Axial length: 22.67 mm, visual field mean deviation: −0.49 dB). (**A**) Retinal nerve fiber layer deviation and thickness-guided progression analysis from Cirrus optical coherence tomography shows stable retinal nerve fiber layer thickness, (**B**) ganglion cell–inner plexiform layer deviation and thickness-guided progression analysis from Cirrus optical coherence tomography shows progressive ganglion cell–inner plexiform layer thickness loss, and (**C**) infrared image of lamina cribrosa from Spectralis optical coherence tomography shows small lamina cribrosa curvature index.

Age, peripapillary choroidal microvascular dropout, systolic and diastolic blood pressure, and the presence of hypertension and diabetes mellitus tended to damage the GCIPL predominantly rather than the RNFL. However, the difference was not statistically significant (Table 1). In addition, the presence of hypertension and diabetes mellitus was more related to the GCIPL-dominant progression group (OR, 3.58; CI = 0.887, 14.439; *p* = 0.0731 and OR, 13.423, CI = 0.619, 291.018; *p* = 0.098, respectively); however, this result also had no statistical significance. A representative case for the GCIPL-dominant progression group, showing progressive GCIPL thickness loss with stable RNFL thickness and relatively smaller LCCI, is illustrated in Figure 2.

## 4. Discussion

Recent advancements in OCT segmentation algorithms have enabled the visualization and measurement of individual retinal layer thickness, including the RNFL and GCIPL. Therefore, this enabled the structure–function relationships to be improved. RNFL defects in glaucomatous damage are well known, and recent studies have demonstrated evidence of early damage to the GCIPL at the macula in some patients with glaucoma [6,7]. Early foveal involvement, which includes GCIPL loss, has been assumed to be more related to factors other than IOP [8,9,10].

This study demonstrated that RNFL-dominant progression was more strongly associated with pre-treatment IOP, mean IOP, and LCCI than GCIPL-dominant progression. Notably, GCIPL progression was associated with factors other than IOP, including pcMvD, systolic and diastolic blood pressure, the presence of hypertension and diabetes mellitus, and age, although the association was not statistically significant. To the best of our knowledge, many studies have proposed a difference in LC architecture based on the IOP level. Thus, these seem to support the biomechanical theory of glaucoma pathogenesis. However, no study has categorized patients with glaucoma into RNFL- or GCIPL-dominant progression groups and compared their associated factors.

To date, elevated IOP has proven to be the principal manageable risk factor for the development and progression of glaucoma [11,12,13]. However, the exact mechanism of how IOP contributes to the glaucomatous damage is not yet completely understood [11,12,13]. The LC has been considered the primary site of IOP-related pathogenesis in glaucoma [14,15,16,17,18,19]. Furthermore, an experimental study in an early glaucoma model revealed that morphologic changes in the LC preceded damage to the RNFL [14]. However, biomechanical changes in the LC are assumed to induce damage to axonal and/or RGCs through various mechanisms, including the blockade of axonal transport and tissue remodeling by reactive astrocytes [20,21,22]. Therefore, LC is considered as the primary site of pressure-related RNFL damage, and this damage can be measured through LCCI [1,23]. Moreover, a cross-sectional study compared the LC structure between patients with high-tension glaucoma and NTG and revealed that the LC in high-tension glaucoma eyes has greater curvature relative to NTG and healthy eyes of similar age [23]. This study also showed that IOP-related glaucomatous damage could be demonstrated through LCCI, and further, that this was more related to RNFL- than GCIPL-dominant progression.

In this study, GCIPL-dominant glaucomatous progression was relatively unrelated to IOP. Although IOP is considered as the most significant risk factor for glaucoma progression, several others, including aberrant systemic and ocular hemodynamics, have also been reported to influence the development and progression of glaucoma. In this study, the GCIPL-dominant progression group had a low LCCI (*p* = 0.031) and tended to have higher systolic and diastolic blood pressure and more pcMvD.

This study had certain limitations: primarily, the small sample size and the relatively short follow-up period. Therefore, due to the small sample size, the statistical validity was low for factors, including pcMvD and blood pressure. Secondly, our study analyzed patients from a single ethnic group. Therefore, when generalizing our results, caution should be exercised. Finally, additional studies with a larger population and longer follow-up periods should be conducted to determine the factors related to each group in diagnosis and to treat patients with glaucoma differently. Nevertheless, this study is meaningful as it is the first to categorize OCT glaucoma patterns into two groups (RNFL- and GCIPL-dominant progression groups) and analyze each associated factor.

## 5. Conclusions

This study categorized glaucomatous damage into the following two different groups: RNFL- and GCIPL-dominant progression groups. The RNFL-dominant progression group was more strongly associated with IOP and LC curvature. However, age, peripapillary choroidal microvascular dropout, systolic and diastolic blood pressures, and the presence of hypertension and diabetes mellitus tended to damage the GCIPL predominantly rather than the RNFL. Our results suggest the possibility of developing better strategies for diagnosing and managing glaucoma. Specifically, it should be possible to classify patients with OAG into two groups, set different treatment targets, and identify different treatment methods in each group.

## Figures and Tables

**Table 1:** Baseline characteristics.

	RNFL-Dominant Progression Group (22 Eyes, 12 Patients)	GCIPL-Dominant Progression Group (36 Eyes, 21 Patients)	*p*-Value
Age [years]	54.95 ± 15.63	62.97 ± 13.58	**0.044**
Female gender (%)	6 (50)	10 (47.62)	0.896
Hypertension [patients]	3	13	0.063
Diabetes mellitus [patients]	0	8	**0.019**
Diagnosis [eyes]			**0.005**
POAG	20 (90.91)	20 (55.56)	
NTG	2 (9.09)	16 (44.44)	
Pre-treatment IOP [mmHg]	19.31 ± 4.45	15.69 ± 3.47	**0.004**
Mean IOP [mmHg]	14.81 ± 1.30	13.46 ± 2.13	**0.006**
Axial length [mm]	25.35 ± 2.21	24.90 ± 1.81	0.486
Visual field MD [dB]	−6.71 ± 6.27	−5.83 ± 5.17	0.566
Glaucoma severity [eyes]			0.350
Early	12 (54.56)	20 (55.56)	
Moderate	4 (18.18)	11 (30.56)	
Severe	6 (27.27)	5 (13.89)	
Presence of pcMvD [eyes]	1 (16.67)	9 (64.29)	0.141
Choroidal thickness [µm]	233.64 ± 78.86	217.92 ± 100.27	0.534
LCCI	27.54 ± 8.06	22.11 ± 9.62	**0.031**
Mean SBP	118.8 ± 14.8	123.7 ± 11	0.151
Mean DBP	71.6 ± 11.8	72.3 ± 7.3	0.806
Presence of disc hemorrhage [eyes]	3	10	0.332

Data are presented as the mean ± standard deviation or n (%). Factors with statistical significance are indicated in bold. Comparisons were performed using *t*-test and Fisher’s exact test. POAG, primary open-angle glaucoma; NTG, normal tension glaucoma; IOP, intraocular pressure; MD, mean deviation; dB, decibels; pcMvD, peripapillary choroid microvascular dropout; LCCI, lamina cribrosa curvature index; SBP, systolic blood pressure; DBP, diastolic blood pressure.

**Table 2:** Factors associated with retinal nerve fiber layer-dominant loss group (logistic regression).

	Univariate			Multivariate Analysis 1			Multivariate Analysis 2		
	OR	95% CI	*p*-Value	OR	95% CI	*p*-Value	OR	95% CI	*p*-Value
Axial length	1.126	0.657–1.580	0.4427						
Age, per 1 year older	**0.962**	**0.925–1**	**0.0493**	0.977	0.929–1.028	0.3738	0.99	0.943–1.04	0.6992
LCCI	**1.070**	**1.004–1.140**	**0.0368**	1.043	0.953–1.141	0.3645	**1.094**	**1.007–1.188**	**0.0345**
Pre-treatment IOP	**1.292**	**1.057–1.580**	**0.0124**	**1.325**	**1.058–1.661**	**0.0143**			
Mean IOP	**1.522**	**1.066–2.053**	**0.019**				**1.655**	**1.139–2.405**	**0.0083**
Choroidal thickness	1.002	0.996–1.008	0.5272						
Visual field test MD	0.972	0.883–1.070	0.5591						
Mean SBP	0.967	0.923–1.013	0.155						
Mean DBP	0.992	0.935–1.052	0.7784						
Hypertension	0.279	0.069–1.127	0.0731						
Diabetes mellitus	0.074	0.003–1.616	0.098						

Statistically significant factors are in bold. RNFL, retinal nerve fiber layer; CI, confidence interval; IOP, intraocular pressure; LCCI, lamina cribrosa curvature index; MD, mean deviation; SBP, systolic blood pressure; DBP, diastolic blood pressure; OR, odds ratio.

## Data Availability

The datasets generated and/or analyzed during the current study are available from the corresponding author upon reasonable request.
